# Long Non-coding RNA MIAT Knockdown Prevents the Formation of Intracranial Aneurysm by Downregulating ENC1 via MYC

**DOI:** 10.3389/fphys.2020.572605

**Published:** 2021-01-21

**Authors:** Xinguo Li, Hang Zhao, Jihui Liu, Jing Tong

**Affiliations:** ^1^Department of Neurosurgery, The First Hospital of China Medical University, Shenyang, China; ^2^Department of Gastroenterology, The First Hospital of China Medical University, Shenyang, China

**Keywords:** intracranial aneurysm, long non-coding RNA MIAT, ectodermal-neural cortex 1, myelocytomatosis oncogene, apoptosis

## Abstract

Intracranial aneurysm (IA) is vascular enlargement occurred on the wall of cerebral vessels and can result in fatal subarachnoid hemorrhage when ruptured. Recent studies have supported the important role of long non-coding RNAs (lncRNAs) in IA treatment. This study identified functional significance of lncRNA myocardial infarction associated transcript (MIAT) in IA. Myocardial infarction associated transcript and ectodermal-neural cortex 1 (ENC1) expression was detected by reverse transcription quantitative polymerase chain reaction. Cell counting kit 8 assay flow cytometry were conducted to detect cell viability and apoptosis of endothelial cells in IA. The interaction among MIAT, ENC1, and myelocytomatosis oncogene (MYC) was analyzed by RNA pull down, RNA immunoprecipitation assay, chromatin immunoprecipitation assay, and dual luciferase reporter assay. Intracranial aneurysm was induced by ligating the left carotid artery and the bilateral posterior branch of the renal artery in rats for studying the role of MIAT and ENC1 *in vivo*. Myocardial infarction associated transcript and ENC1 were upregulated in IA. Endothelial cells in IA presented a decreased cell viability and an increased apoptotic rate. Myocardial infarction associated transcript could regulate the expression of ENC1, and MYC could bind to the promoter region of ENC1. High expression of MIAT increased endothelial cell apoptosis and vascular endothelial injury, while MIAT knockdown was identified to reduce the risk of IA both *in vitro* and *in vivo* through regulating ENC1. To sum up, MIAT silencing is preventive for IA occurrence by decreasing the MYC-mediated ENC1 expression, which represents a novel therapeutic target for IA.

## Introduction

Intracranial aneurysm (IA) is a life-threatening disorder leading to subarachnoid hemorrhage with an incidence of approximately 1–2%. Intracranial aneurysm rupture accounts for 30–40% of IA-related mortality (Aoki et al., 2019; Fan et al., 2019; Li et al., 2019). The conventional treatment protocols for IA include surgical clipping and endovascular coiling (Walcott et al., 2016). In addition, flow disruption has been reported is an emerget endovascular protocol for IA management (Piotin et al., 2018). Nevertheless, various complications like coil compaction or aneurysm growth leading to IA recurrence arise after endovascular treatment (Abdihalim et al., 2014). Therefore, an extensive exploration of the underlying mechanisms of IA pathogenesis is of vital significance for improving the prognosis of IA (Miyata et al., 2017).

Essentially, long non-coding RNAs (lncRNAs), which are RNA molecules with over 200 nucleotides and with constrained protein-coding ability (Qi et al., 2016), are involved in IA formation by regulating various pathways associated with immune or inflammatory processes and vascular smooth muscle contraction (Li et al., 2017). Of note, initial bioinformatic analysis of this study identified differentially expressed lncRNA myocardial infarction associated transcript (MIAT) related to neurovascular disease. Existing evidence has suggested the potential of MIAT to modulate the endothelial cell function via interactions with vascular endothelial growth factors (Yan et al., 2015). Myocardial infarction associated transcript has been highlighted to accelerate diabetes-induced microvascular dysfunction (Yu and Wang, 2018). Furthermore, abnormal expression of MIAT has been identified in the context of neurovascular dysfunction, and silencing of MIAT could evidently result in cerebral microvascular degeneration (Jiang et al., 2016). Although an investigation on whether MIAT participated in the formation of IA is warranted, we speculated an association between MIAT and the underlying pathogenesis for the formation and rupture of IA.

Furthermore, we predicted that MIAT was involved in the pathogenesis of IA *via* interaction with ectodermal-neural cortex 1 (ENC1) and transcription factor (TF) myelocytomatosis oncogene (MYC). Ectodermal-neural cortex 1, as a member of the kelch family, is a vital modulator in the process of neuronal differentiation (Lei et al., 2016). Moreover, a recent study revealed the potential of ENC1 as a favorable candidate for ovarian cancer treatment (Fan et al., 2019). Myelocytomatosis oncogene is an oncoprotein that mediates the expression of genes correlated with metabolism, differentiation and survival in malignancies (Yun et al., 2019). Also, further evidence has indicated that the transcription activity of MYC can be controlled by lncRNAs such as lncRNA gastric cancer-associated lncRNA1 and lncRNA plasmacytoma variant translocation 1 (Jin et al., 2019b; Zhuang et al., 2019). Our study was performed to clarify the functional relevance of MIAT in IA, which was involved with the interaction with ENC1 and MYC. The findings in our study may shed new light to broaden the understanding of IA and to develop novel therapeutic modalities to prevent the formation and rupture of IA.

## Materials and Methods

### Ethics Statement

The current study was performed with approval of the Ethics Committee of the First Hospital of China Medical University. All participants provided signed informed consent documentation prior to enrollment. All animal experiments were performed in strict accordance with the recommendations issued in the Guide for the Care and Use of Laboratory Animals of the National Institutes of Health.

### Microarray Based Analysis

IA-related microarray GSE75436 was adopted from the Gene Expression Omnibus database,^1^ and the ‘‘limma’’ package^2^ in R software was used for conducting differential analysis with | log foldchange (FC)| > 2 and *p* < 0.01 as the screening threshold. The data of the human lncRNAs were downloaded from lncMAP, after which the Venn diagram of the differential expression analysis and lncRNA data was plotted, and the differentially expressed lncRNAs in GSE75436 were screened as the key lncRNAs. The localization of the selected lncRNA and its regulatory mechanism were predicted using the lncATLAS.^3^ The LncRNA-TF-Gene predictive ability of LncMAP^4^ was employed to predict the downstream TFs that were regulated by the selected lncRNAs. The protein--protein interaction (PPI) analysis of predicted TFs was conducted on String,^5^ the results of which were processed using Cytoscape^6^ to screen the key TFs. Then the intersection between the differentially expressed genes in microarray GSE75436 and the downstream genes of key TFs was taken, the Venn diagram was plotted to screen out the downstream gene, after which the regulatory pathway with higher credibility was predicted.

### Study Subjects

A total of 85 patients diagnosed with IA in the First Hospital of China Medical University from May 2010 to May 2012 were enrolled in the study, among them, there were 38 documented cases of unruptured IA and 47 cases of ruptured IA. The size of the aneurysm ranged from 20 mm × 15 mm to 2.5 mm × 1.5 mm. Specimens of the same artery were obtained as control samples from 18 patients with brain tumor (*n* = 11) and subacute subdural hematoma (*n* = 7) treated by peritoneal craniotomy (non-aneurysm or vascular malformation pathology). The last follow-up was on October 30, 2018, and the average observation time for survivors was 60 months (9–78 months). No significant differences were evident in parameters such as age and gender among the participants.

### Culture of Endothelial Cells

Vascular endothelial cells HBEC-5i (ATCC^®^ CRL-3245^TM^) were cultured using Dulbecco’s modified eagle’s medium (DMEM, F12, ATCC^®^ 30-2006^TM^) containing 40 μg/mL of endothelial cell growth supplement and 10% fetal bovine serum (FBS). Endothelial cells were transduced separately: overexpression (oe)-negative control (NC) + short hairpin RNA (sh)-NC group (transduced with both overexpression and shRNA empty plasmids), oe-MIAT + sh-NC group (transduced with MIAT overexpression plasmid + shRNA empty plasmid), oe-NC + sh-ENC1 group (transduced with overexpression empty plasmid + shRNA-ENC1 plasmid), oe-MIAT + sh-ENC1 group (transduced with MIAT overexpression plasmid + shRNA-ENC1 plasmid).

### Construction and Transduction of Lentiviral Vectors

The 293T cells (ATCC^®^ ACS-4500^TM^) were cultured using DMEM containing 10% FBS, 1% penicillin and streptomycin at 37°C under 5% CO_2_. Lentiviral packaging plasmids (MIAT overexpression, sh-MIAT, sh-ENC1, and sh-NC plasmids) were purchased from GenePharma (Shanghai, China). Cells in the logarithmic growth phase were trypsinized and seeded into 24-well plates, which were subsequently cultured into the monolayer cells. The culture medium was discarded. The cells were transduced separately: the sh-NC group (transduced with shRNA empty plasmid), the oe-NC group (transduced with overexpression empty plasmid), the sh-MIAT group (transduced with shRNA-MIAT plasmid), the oe-MIAT group (transduced Transduction with MIAT overexpression plasmid) and the sh-ENC1 group (transduction with shRNA-ENC1 plasmid). Cells were seeded in six-well plates 24 h before transduction. Upon attaining 30–50% cell confluence, the cells were transduced in strict accordance with the provided instructions of lipofectamin 2000 (11668-019, Invitrogen, Carlsbad, CA, United States). Next, 4 μg of plasmid was diluted using 250 μL of serum-free medium Opti-MEM (51985042, Gibco, Gaitherburg, MD, United States) (final concentration = 50 nM) and incubated at room temperature for 5 min. Then, 5 μL lipofectamin 2000 was diluted using 250 μL of serum-free medium Opti-MEM and incubated at room temperature for 5 min. The aforementioned solutions were mixed together and incubated at room temperature for 20 min before the addition of the cell culture wells. Cells from each group were incubated at 37°C and 5% CO_2_ for 6–8 h. The medium underwent complete medium replacement, and the cells were cultured for 24–48 h before subsequent experiments.

### Construction of a Rat Model of IA

The model of IA was established in male Sprague Dawley (SD) rats, aged 7 weeks. A total of 36 rats were randomly classified into four groups: the sham group (*n* = 12, IA model rats without virus injection), the IA + sh-NC group (*n* = 12, IA model rats injected with sh-NC packaged virus), the IA + sh-MIAT group (*n* = 6, IA model rats injected with sh-MIAT packaged virus), and the IA + sh-ENC1 group (*n* = 6, IA model rats injected with sh-ENC1 packaged virus). In brief, the rats were anesthetized using an intraperitoneal injection of pentobarbital (50 mg/kg), and the left carotid artery and the bilateral posterior branch of the renal artery were ligated to increase the hemodynamic stress at the bifurcation site of the contralateral carotid artery. Rats were instilled a high salt diet containing 8% sodium chloride and 0.12% 3-aminopropionitrile in order to induce systemic hypertension by salt overdose. Rats were granted free access to food after successful induction of IA.

According to the information of MIAT and ENC1 in National Centre for Biotechnology Information (NCBI) database, lentiviral vectors of sh-MIAT and sh-ENC1 were designed and packaged. In this experiment, the construction, identification and sequencing of lentiviral vectors for MIAT and ENC1 genes, plasmid extraction and packaging of lentiviruses were all completed by Shanghai SunBio Biomedical Technology Co., Ltd. (Shanghai, China). The lentiviral vectors were co-transfected into 293T cells for acute viral packaging, and the cell supernatants were collected after 72 h of culture at 37°C with 5% CO_2_. The cell supernatant was filtered with a 0.45 μm filter, and then the filtrate was ultracentrifuged at 4°C and 20,000 rpm for 2 h. The obtained concentrated virus liquid was sub-packed into test tubes. The 293T cells were re-infected with diluted packaged viral fluid. The lentiviral packaging plasmids of sh-NC, sh-MIAT or sh-ENC1 sequences homologous to human and rat were transfected into 293T cells using Lipofectamine 2000 (Invitrogen), respectively. After 48 h, viral particles were collected and injected intraperitoneally into each rats at 1 × 10^8^ transduction units every day after surgical induction of IA, repeated weekly for 4 weeks. On the 30th day, mice were anesthetized by intraperitoneal injection of 50 mg/kg phenobarbital, followed by collection of intracranial cerebral aneurysm tissues. The tissue was fixed in 4% paraformaldehyde solution at 4°C for 24 h, then dehydrated by conventional gradient alcohol, and embedded in paraffin. Serial coronal sections were made at the optic chiasm with a thickness of 4 μm (some sections were prepared for hematoxylin-eosin (HE) staining and TUNEL detection of apoptosis). The shRNA sequences are depicted in Table 1.

**TABLE 1:** The shRNA sequences.

shRNAs	Sequence (DNA) (5′–3′)
sh-MIAT	GGATCCACTTTATGTGGATGT
sh-ENC1	GGATGTGACTTACATTGTTGA
sh-NC	CAGATATGCCAATGCTAGGA

*MIAT, myocardial infarction associated transcript; ENC1, ectodermal-neural cortex 1; sh/shRNA, short hairpin RNA; NC, negative control.*

### Nuclear and Cytoplasmic Fractionation

Separation of nucleus and cytoplasm of vascular endothelial cells HBEC-5i was conducted using PARIS^TM^ Kit (Invitrogen^TM^, AM1921) according to the operation steps of the instructions, followed by reverse transcription quantitative polymerase chain reaction (RT-qPCR) to detect MIAT expression in different cell components.

### RNA Isolation and Quantitation

Total RNA was extracted from cells and tissue samples using the TRIzol kit (cat: 15596018; Invitrogen, Carlsbad, CA, United States), after which the RNA concentration was determined. RT was conducted using the cDNA RT kit (K1622, Beijing Yaanda Biotechnology Co., Ltd., Beijing, China). Genomic DNA was extracted from blood samples using a DNA purification kit [FlexiGene DNA kit (Qiagen, Hilden, Germany) or Oragene^®^ DNA sample collection kits (DNA Genotek, ON, Canada)]. Next, RT-qPCR was conducted in accordance with the pre-mixed PreTaq II kit (Takara, Dalian, Liaoning, China) of Synergy Brands. β-actin served as the internal reference and the primers used are presented in Table 2.

**TABLE 2:** Primer sequence for RT-qPCR.

Gene	Forward (5′–3′)	Reverse (5′–3′)
MIAT	TATTTGCAGGGGGTGCTCTG	GGGCAGGGGGTCTAACTCTA
MYC	CCTACCCTCTCAACGACAGC	CTCTGACCTTTTGCCAGGAG
ENC1	CCACTGCTCAGCGTCTCTTC	TTTCAGGCCACCACTGAACA
β-actin	ACGACATGGAGAAGATCTG	TGTTGAACGTCTCGAACATG

*MIAT, myocardial infarction associated transcript; MYC, myelocytomatosis oncogene; ENC1, ectodermal-neural cortex 1; RT-qPCR, reverse transcription quantitative polymerase chain reaction.*

### Western Blot Analysis

Total protein was isolated from the endothelial cells using radioimmunoprecipitation assay (RIPA) lysis containing phenylmethylsulfonyl (R0010, Solarbio, Beijing, China). Then 50 μg of protein was subjected to 10% sodium dodecyl sulfate (SDS)-polyacrylamide gel electrophoresis, and then transferred onto a polyvinylidene fluoride (PVDF) membrane. The membrane was then incubated with the following diluted primary antibodies: rabbit primary polyclonal antibody to ENC1 (1: 1000, ab106683), rabbit monoclonal antibody to MYC (1: 1000, ab32072), rabbit polyclonal antibody to cleaved Caspase-3 (1: 500, ab49822), rabbit anti-cleaved Poly-(ADP-ribose) polymerase 1 (PARP1) (1: 2000, 44-698G), rabbit monoclonal antibody to B-cell lymphoma-2 (Bcl-2) associated protein X (Bax) (1: 10,000, ab32503), rabbit polyclonal antibody to Bcl-2 (1: 2000, ab196495), and murine monoclonal antibody to β-actin (1: 10,000, ab8226). The membrane underwent overnight incubation with the aforementioned antibodies at 4°C, and after three rinses using the *Tris* Buffered Saline with Tween, the membrane was incubated with the horseradish peroxidase-labeled secondary antibody immunoglobulin G (IgG) (ab205718, goat anti-rabbit, 1: 20,000; ab205719; goat anti-mouse 1: 20,000) for 1 h. The above-mentioned antibodies were purchased from Abcam Inc., (Cambridge, MA, United States) except for the rabbit anti-cleaved PARP1 (Invitrogen). Enhanced chemiluminescence Fluorescence Detection Kit (Cat. No. BB-3501, Amersham, Little Chalfont, United Kingdom) was used for appropriate exposure and imaging. The Bio-Rad image analysis system (BIO-RAD, Hercules, CA, United States) was employed for photography and Quantity One v4.6.2 software was adopted for analysis. The relative protein content was expressed as an equivalent of the gray value of the corresponding protein band to the gray value of the β-actin (internal reference) protein band.

### Dual-luciferase Reporter Assay

The ENC1 promoter region was cloned into the pmirGLO luciferase vector (Promega, Madison, WI, United States) to construct the pGL4.16-ENC1 prom wild type (wt) (ENC1 prom wt) vector and pGL4.16-ENC1 prom mutant type (mut) (ENC1 prom mut) vector. Cell transfection was performed in accordance to the Lipofectamine 2000 instructions. 293T cells were transduced with sh-NC + oe-NC, sh-MIAT + oe-NC, sh-NC + oe-MYC, or sh-MIAT + oe-MYC, respectively, in combination with ENC1 prom wt and ENC1 prom mut. The Renilla luciferase expression plasmid pRL-TK (TaKaRa, Dalian, Liaoning, China) served as the internal reference. Cells were cultured for 24 h after transfection, and then the dual luciferase activity was detected in compliance with the Dual-Luciferase Reporter Assay System (Promega, Madison, WI, United States).

### Hematoxylin-eosin Staining

Arterial wall of patients with IA was fixed using 4% paraformaldehyde, dehydrated, embedded in paraffin, and conventionally sectioned (thickness = 3–4 μm). HE staining was conducted to observe the pathological changes of the arterial wall. Paraffin sections were heated in an oven at 80°C for 1 h. After the sections were cooled, they were dehydrated using gradient alcohol, cleared using xylene, and rinsed under tap water. The sections were stained using hematoxylin (H8070-5g, Solarbio, Beijing, China) for 4 min, and hydrolyzed with hydrochloric acid-ethanol for 10 s. The sections were re-blued using ammonia for 10 min. Next, the sections were stained with eosin solution (PT001, Shanghai Bogoo Biotechnology, Co., Ltd., Shanghai, China) for 2 min. The sections were dehydrated using gradient alcohol and cleared with xylene. The sections were sealed using neutral gum and the pathological changes in the arterial wall of patients were observed under an optical microscope (DMM-300D, Shanghai Caikon Optical Instrument Co., Ltd., Shanghai, China).

### Immunofluorescence Staining

Paraffin sections of arterial wall from IA patients were dewaxed three times using xylene and then hydrated with gradient ethanol. Antigen retrieval was performed using 0.01 mol/L sodium citrate buffer at 98°C for 20 min. The sections were cooled to room temperature and cleared using 0.1% Triton X 100 for 5 min. Sections were blocked with 10% goat serum for 60 min at room temperature, and then incubated with the primary antibody: rabbit anti-human CD34 (ab228540, 1: 200, Abcam Inc., Cambridge, MA, United States) at 4°C overnight. The excess primary antibody was rinsed using 1% BSA. Next, the sections were incubated with the goat anti-rabbit secondary antibody (ab6717, 1: 1000, Abcam Inc., Cambridge, MA, United States) at room temperature for 60 min in conditions devoid of light. The excess secondary antibody was washed off with 1% BSA in the dark. The sections were observed and photographed under a fluorescence microscope.

### RNA Pull-down Assay

Myocardial infarction associated transcript labeled with *in vitro* transcription synthesized biotin was incubated with the endothelial cell extracts, which subsequently underwent incubation with the streptavidin agarose beads (Thermo Fisher Scientific, Waltham, MA, United States). The complex was eluted after washing. The protein product complexes were subjected to Western blot analysis.

### RNA Immunoprecipitation Assay

Epithelial cells were lysed using RIPA lysis buffer containing protease inhibitor and RNase inhibitor [10 mM Hepes (pH 7.5), 150 mM KCl, 0.5% Nonidet P-40, 2 mM ethylenediaminetetraacetic acid, 1 mM NaF], and centrifuged at 16,400 × *g* for 10 min. Then, the cell lysate was incubated with the primary monoclonal antibody to MYC (ab32072, Abcam Inc., Cambridge, MA, United States) or anti-rabbit IgG (ab205718, goat anti-rabbit, 1: 20,000, Abcam Inc., Cambridge, MA, United States) at 4°C for 3 h. Then the cells were incubated with 30 μL of protein G magnetic beads (Invitrogen, Carlsbad, CA, United States) for 3 h at 4°C. The cells were rinsed three times using the RIP wash buffer [50 mM Hepes (pH 7.5), 150 mm KCl, 0.05% Nonidet P-40] containing RNase inhibitor (Promega, Madison, WI, United States). The RNA was extracted using a total RNA isolation kit (Macherey-Nagel, Düren, Germany) and measured by conducting RT-qPCR.

### Chromatin Immunoprecipitation Assay

After cross-linking the cells using 4% formaldehyde, the cells were disrupted by exposure to ultrasonic waves to obtain 200–1000 bp DNA–protein complex. Myelocytomatosis oncogene antibody was added to harvest DNA–MYC complex targeted by MYC antibody. DNA–MYC complex was incubated with Protein A Agarose/SaLmon Sperm DNA was supplemented to bind to MYC antibody-MYC-ENC1 promoter complex. These complexes were precipitated and rinsed to remove non-specific binding. The enriched MYC-ENC1 complexes were isolated and de-crosslinked. The enriched ENC1 promoter fragments were subjected to RT-qPCR analysis after purification.

### Cell Counting Kit 8 Assay

Vascular endothelial cells were subjected to culture in a 96-well plate for 24 h at 37°C with 5% CO_2_. The cells were incubated for another 24 h after transfection with oe-MIAT or oe-NC. Then each well was incubated with 10 μL of cell counting kit 8 (CCK-8) solution for 4 h. The absorbance value (A) of each well was measured using a multi-functional microplate reader at an excitation wavelength of 450 nm. Cell survival rate = (experimental absorbance value/control well absorbance value) × 100%. The experiments were repeated three times independently.

### Terminal Deoxynucleotidyl Transferase (TdT)-mediated Deoxyuridine Triphosphatase (dUTP)-Biotin Nick End Labeling (TUNEL) Staining

Arterial wall sections were stained using the fluorescein-coupled TUNEL *in situ* cell death assay kit (Roche, Basel, Switzerland). Briefly, the sections were dewaxed using conventional xylene, hydrated with gradient alcohol, incubated with 20 mg/mL proteinase K for 15 min at room temperature, and immersed in phosphate buffered saline (PBS) solution containing 0.5% H_2_O_2_ at room temperature for 20 min to incapacitate endogenous peroxidase, followed by reaction in equilibrium buffer for 10 min at room temperature. After washing, the sections were incubated with 50 μL of TUNEL at 37°C for 1 h. After mounting the sections with the anti-fade mounting medium, the fluorescence intensity was observed under a fluorescence microscope. The number of TUNEL-positive cells was counted under five randomly selected high-power fields (Song et al., 2018).

### Flow Cytometry

Forty-eight hours after transfection, 1 × 10^6^ endothelial cells were stained with Annexin-V-fluorescein isothiocyanate (Annexin V-FITC) (KeyGEN Bio-tech Co., Ltd., Nanjing, Jiangsu, China) for 15 min at room temperature in conditions devoid of light. Next, the cells were centrifuged at 1000 rpm for 5 min and resuspended using 0.5 mL of pre-cooled binding buffer. Samples were analyzed by a flow cytometer (Bio-Rad Inc., Hercules, CA, United States) after the addition of 10 μL of propidium iodide (PI).

### Statistical Analysis

The SPSS 21.0 statistical software (IBM Corp., Armonk, NY, United States) was adopted for statistical analysis. When conforming to normal distribution and homogeneity of variance, the measurement data were expressed as mean ± standard deviation. Comparisons between two groups were analyzed by the unpaired *t* test and comparisons between multiple groups were analyzed by one-way analysis of variance (ANOVA), followed by Tukey’s *post hoc* test. Pearson correlation analysis was employed to analyze the correlation between ENC1 expression and MIAT expression in patients with IA. Kaplan–Meier analysis was employed to analyze the relationship between the high and low expression of MIAT and ENC1 in patients with IA and the disease-free survival and total survival (log-rank test). A value of *p* < 0.05 was considered to be statistically significant.

## Results

### Patients With IA Presented a Higher Expression of MIAT and Damaged Endothelial Cell Continuity

**FIGURE 1:**
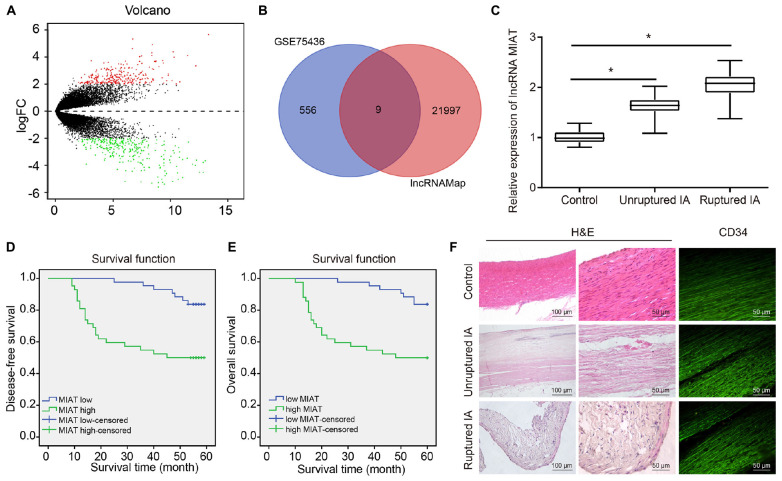
The high expression of MIAT and damaged endothelial cell continuity are observed in patients with IA. **(A)** The volcano plot of GSE75436 analyzed by R language. Red dots represented the upregulated genes, and green dots represented the downregulated genes (LogFC > 2, *p* < 0.01). **(B)** The Venn diagram of the differentially expressed lncRNAs in GSE75436 and the total lncRNAs obtained from lncMAP, and the intersections included lncRNA SFTA1P, CTD-2165H16.3, NAPSB, RP3-525N10.2, CTB-12O2.1, FLVCR2, EMX2OS, EGFEM1P, and MIAT. **(C)** The expression of MIAT in ruptured IA, unruptured IA, and control samples determined by RT-qPCR. **(D)** The correlation between the expression of MIAT and disease-free survival of patients with IA. **(E)** The correlation between the expression of MIAT and overall survival of patients with IA. **(F)** The HE staining (×100, ×200) for carotid arteries near branch or IA lesions, and immunofluorescence staining (×200) for vascular endothelial cells labeled with CD34 antibody. *p < 0.05 vs. the control samples. The measurement data were expressed as mean ± standard derivation. Comparisons between two groups were analyzed by unpaired *t* test, while the correlation between the expression of MIAT in patients with IA and their disease-free survival and overall survival was analyzed by Kaplan–Meier method (long-rank test).

To explore the key lncRNAs that promote IA, analysis of the microarray dataset GSE75436 by R language revealed the presence of 267 genes highly expressed and 298 genes poorly expressed in IA (Figure 1A). Besides, nine differentially expressed lncRNAs were obtained from the Venn diagram (Figure 1B), among them, MIAT was selected for subsequent experimentation as it had been previously identified as a potential pharmacological target for the treatment of neurovascular diseases (Jiang et al., 2016) that MIAT was associated with the pathogenesis underlying the formation and rupture of IA. The blood samples from patients with IA were collected, and the expression of MIAT in patients with ruptured IA, patients with unruptured IA and control samples was determined by RT-qPCR. An increased expression of MIAT was observed in IA, with a more significant increase in ruptured IA, relative to control samples (Figure 1C). Kaplan–Meier analyses showed that ruptured IA patients with high MIAT expression exhibited reduced disease-free survival (Figure 1D) and overall survival (Figure 1E) compared with IA patients with low MIAT expression, presenting with a poor prognosis.

Arterial wall sections were obtained from ruptured IA samples, unruptured IA samples and control samples, followed by HE staining of vascular wall lesions. Results showed that the shape of vascular wall was irregular in patients with IA and were more irregular in patients with unruptured IA. Immunostaining of the CD34 glycoprotein, the results presented that the vascular endothelial cells of control samples were closely connected and arranged neatly along the cavity, while the endothelial cells of patients with IA were absent and disintegrated. Cell destruction was more severe in patients with unruptured IA (Figure 1F).

### Overexpressing MIAT Induced the Apoptosis of Endothelial Cells

**FIGURE 2:**
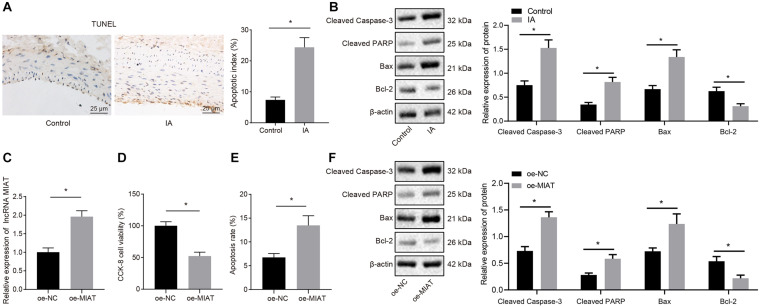
The endothelial cell apoptosis can be induced by overexpression of MIAT *in vitro*. **(A)** Representative images of TUNEL staining (×200) and quantitative analysis of TUNEL-positive cells in patients with IA. **(B)** The expression of cleaved Caspase-3, cleaved PARP1, Bax, and Bcl-2 in patients with IA measured by Western blot analysis. **(C)** The expression of MIAT in endothelial cells after overexpressing MIAT determined by RT-qPCR. **(D)** Viability of endothelial cells in response to oe-MIAT detected by CCK-8 assay. **(E)** Flow cytometry analysis of apoptosis of endothelial cells in response to oe-MIAT. **(F)** The expression of apoptosis-related factors (cleaved Caspase-3, cleaved PARP1, Bax, and Bcl-2) in response to oe-MIAT measured by Western blot analysis. *p < 0.05. The measurement data were expressed as mean ± standard derivation. Comparisons between two groups were analyzed by unpaired *t* test, and the cell experiment was conducted three times independently.

It has been reported that the pathogenesis and development of aneurysms are associated with cell apoptosis (Pentimalli et al., 2004). Hence TUNEL staining was conducted to assess the TUNEL positive rate in the endothelial cells of arterial wall sections in patients with IA. It was found that patients with IA presented increased TUNEL positive rate relative to control samples (Figure 2A), which may result in loss of vascular endothelial cells, and consequently damage the vascular continuity. Consistent with TUNEL staining, Western blot analysis displayed that the expression of cleaved Caspase-3, cleaved PARP1, and Bax was increased, and the Bcl-2 expression was reduced in patients with IA, indicating the enhanced endothelial cell apoptosis in patients with IA (Figure 2B).

Next, the effect of high expression of MIAT on the apoptosis of endothelial cells (HBEC-5i) in IA was explored at cell level. We overexpressed MIAT in vascular endothelial cells and confirmed the overexpression efficiency by RT-qPCR (Figure 2C; *p* < 0.05). Then CCK-8 assay (Figure 2D) and flow cytometry (Figure 2E) depicted that endothelial cell viability was lowered but cell apoptosis was elevated after overexpression of MIAT. Besides, Western blot analysis revealed that the expression of cleaved Caspase-3, cleaved PARP1, and Bax was enhanced, while Bcl-2 expression was diminished after MIAT overexpression (Figure 2F). These results suggest that endothelial cell apoptosis could be induced after overexpression of MIAT, which were consistent with the results of the tissue examination.

### MIAT Upregulated ENC1 Expression through MYC

**FIGURE 3:**
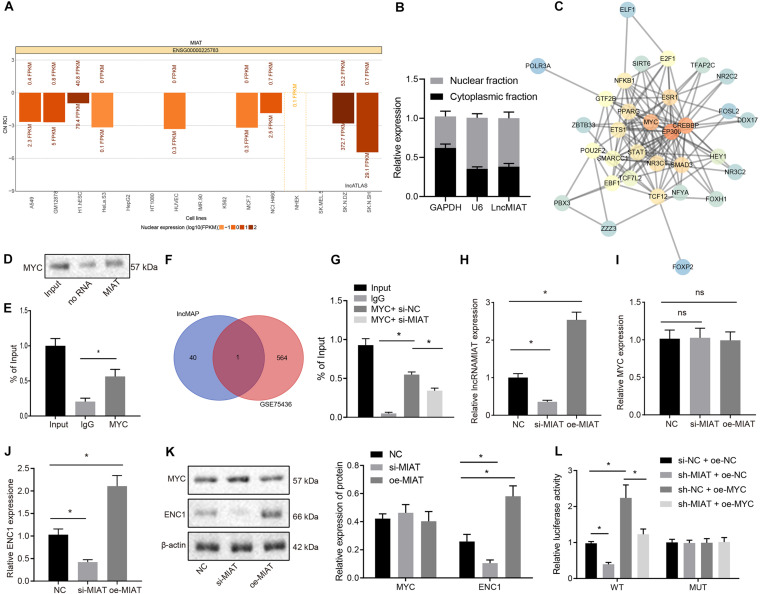
MIAT upregulates ENC1 expression through MYC. **(A)** The subcellular localization of MIAT. CN RCI < 0 indicates that lncRNA is expressed in the nucleus, and CN RCI > 0 indicates that lncRNA is expressed in the cytoplasm. **(B)** After the nucleus and cytoplasm of HBEC-5i cells were separated, MIAT expression was detected by RT-qPCR. GAPDH was a marker of cytoplasm and U6 was a marker of nucleus. **(C)** The downstream TFs of MIAT analyzed by PPI analysis. The redder the color, the higher the core degree was Vice versa, the bluer the color, the lower the core degree was. **(D)** RNA pull down to detect the binding of MYC with MIAT. **(E)** RIP to detect the binding of MYC with MIAT. **(F)** The Venn map of the differentially expressed genes in the microarray GSE75436 and the downstream genes of TF MYC, and the intersection was ENC1. **(G)** The enrichment of MYC binding to the promoter region of ENC1 by ChIP. **(H)** MIAT expression in endothelial cells after overexpressing or silencing MIAT detected by RT-qPCR. **(I)** MYC mRNA expression in endothelial cells after overexpressing or silencing MIAT detected by RT-qPCR. **(J)** ENC1 expression in endothelial cells after overexpressing or silencing MIAT detected by RT-qPCR. **(K)** Protein expression of MYC and ENC1 in endothelial cells after overexpressing or silencing MIAT determined by Western blot analysis. **(L)** The regulatory effect on ENC1 by MIAT and MYC detected by dual luciferase reporter gene assay. *p < 0.05, ns *p* > 0.05. The measurement data were expressed as mean ± standard derivation, comparisons among multiple groups were analyzed by one-way ANOVA and followed by Tukey’s *post hoc* test, and the cell experiment was conducted three times independently.

Through the expression map obtained by lncALAS (Figure 3A), it was found that MIAT was primarily localized in the nucleus. In addition, we isolated the nucleus and cytoplasm of HBEC-5i, which also verified the expression of MIAT in the nucleus (Figure 3B). Totally, 33 TFs and 523 LncRNA-TF-Gene regulatory pathways were obtained based on the data from a similar brain disease low-grade glioma predicated by lncMAP. Protein–protein interaction analysis of TFs identified the top three TFs regarding the core degree, which were EP300, CREBBP, and MYC (Figure 3C). Myelocytomatosis oncogene was proven to be involved in angiogenesis (Yun et al., 2017; Spuler, 2019). To verify whether there is an interaction between MIAT and MYC, the *in vitro* transcriptional synthetic biotin-labeled MIAT was incubated with the extract of vascular endothelial cells in RNA pull down assay to enable MIAT to interact with proteins. Western blot analysis demonstrated the ability of MYC to bind to MIAT (Figure 3D). Consistently, RIP assay was applied to detect MYC-binding RNA in endothelial cells, and RT-qPCR in precipitated samples detected the enrichment of MIAT (Figure 3E), suggesting that MIAT could interact with MYC.

Next, 41 downstream genes of the MIAT-MYC-Gene regulatory pathway were screened out, that is, 41 downstream genes of the TF MYC. An intersection was taken between these genes and the differentially expressed genes obtained in the microarray GSE75436, and ENC1 was obtained from the Venn map (Figure 3F). Since ENC1 was associated with various cancers and brain diseases, we intended to investigate the existence of an association between ENC1 and IA (Fujita et al., 2001; Lee et al., 2016; Fan et al., 2019).

After silencing or overexpression of MIAT in the endothelial cells, RT-qPCR (Figures 3H–J) and Western blot analysis (Figure 3K) displayed that silencing or overexpression of MIAT did not affect MYC expression but could regulate the ENC1 expression. Chromatin immunoprecipitation (ChIP) assay revealed that MYC could bind to the promoter region of ENC1 and silencing MIAT caused the decline of the binding (Figure 3G). Furthermore, dual luciferase reporter assay revealed that silencing MIAT reduced the luciferase activity of ENC1-prom-WT, which was restored by MYC, whereas there was no significant difference in luciferase activity among each group after mutation of the binding site sequence (Figure 3L). Conjointly, MIAT binds to MYC, thereby upregulating ENC1 expression.

### ENC1 Expression Was Elevated in IA Patients, and si-ENC1 Reversed MIAT-induced Vascular Endothelial Cell Apoptosis

**FIGURE 4:**
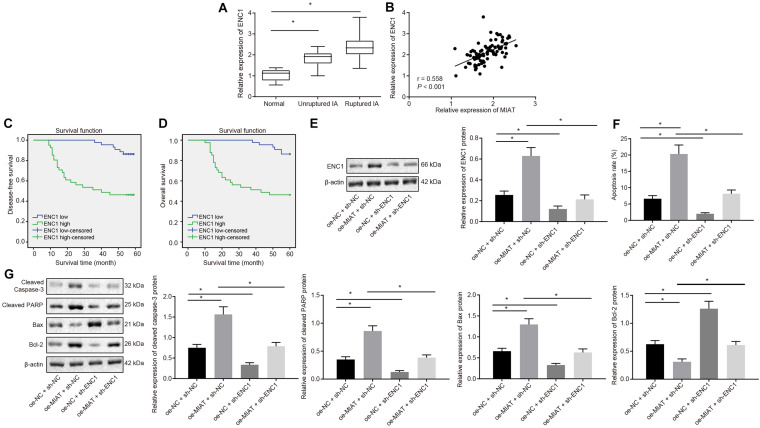
ENC1 expression is augmented in IA patients, and ENC1 silencing negates MIAT-induced endothelial cell apoptosis. **(A)** ENC1 expression in ruptured IA, unruptured IA and control samples determined by RT-qPCR. **(B)** Analysis of the correlation between ENC1 expression and MIAT expression. **(C)** The correlation between the expression of ENC1 and disease-free survival of patients with IA. **(D)** The correlation between the expression of ENC1 and overall survival of patients with IA. **(E)** The protein expression of ENC1 in vascular endothelial cells *in vitro* in response to oe-MIAT and sh-ENC1 alone or in combination measured by Western blot analysis. **(F)** Flow cytometry analysis for apoptosis of endothelial cells in response to oe-MIAT and sh-ENC1 alone or in combination. **(G)** The expression of apoptosis-related factors (cleaved Caspase-3, cleaved PARP1, Bax, and Bcl-2) in response to oe-MIAT and sh-ENC1 alone or in combination measured by Western blot analysis. *p < 0.05. The measurement data were expressed as mean ± standard derivation. Comparisons between two groups were analyzed by unpaired *t* test, while the correlation between the expression of ENC1 in patients with IA and their disease-free survival and overall survival was analyzed by Kaplan–Meier method (long-rank test). Comparisons among multiple groups were analyzed by one-way ANOVA followed by Tukey’s *post hoc* test, and the cell experiment was conducted three times independently.

To explore whether ENC1 acts as a downstream target gene of the MIAT–MYC axis to orchestrate MIAT-induced vascular endothelial cell apoptosis, RT-qPCR assay revealed that ENC1 expression was significantly elevated in IA patients compared to the normal control subjects. In IA patients, ENC1 expression was significantly increased in patients with ruptured IA compared to patients with unruptured IA (Figure 4A). Moreover, ENC1 expression was positively correlated with MIAT expression (Figure 4B). Kaplan–Meier analyses showed that ruptured IA patients with high ENC1 expression exhibited reduced disease-free survival (Figure 4C) and overall survival (Figure 4D) compared to the IA patients with low ENC1 expression, presenting with a poor prognosis.

Vascular endothelial cells were cultured *in vitro*. Results of Western blot showed analysis described that ENC1 expression was significantly elevated in the presence of oe-MIAT and diminished in the presence of sh-ENC1, which was neutralized in response to oe-MIAT + sh-ENC1 (Figure 4E). Flow cytometry (Figure 4F) documented that the apoptosis rate of vascular endothelial cells was significantly increased by oe-MIAT + sh-NC (6.12% vs. 20.36%, *p* < 0.05), but was diminished by oe-NC + sh-ENC1 (6.12% vs. 3.84%, *p* < 0.05), which was normalized by the oe-MIAT + sh-ENC1 treatment (6.12% vs. 8.31%). Simultaneously, Western blot analysis provided consistent results (Figure 4G). The treatment with oe-MIAT resulted in elevated cleaved caspase 3, cleaved PARP1, and Bax, but diminished Bcl-2 expression, and sh-ENC1 led to opposite results. Furthermore, the oe-MIAT + sh-ENC1 treatment annulled these trends. Taken together, knockdown of ENC1 reversed MIAT-induced apoptosis in endothelial cells.

### Silencing of MIAT Inhibited Endothelial Cell Apoptosis and Maintained Endothelial Cell Continuity in IA *in vivo*

**FIGURE 5:**
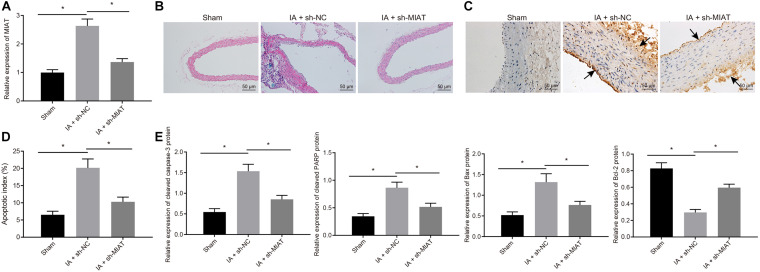
MIAT silencing inhibits endothelial cell apoptosis and maintains endothelial cell continuity in IA rats. Rats were induced with IA model and treated with sh-MIAT or sh-NC, with the sham-operated rats as control. **(A)** The expression of MIAT in rats determined by RT-qPCR. **(B)** HE staining (× 200) for the ACA/OA branch sections of rats. **(C)** The representative images of the IA wall of ACA/OA branch of rats (×200) (arrows indicate the arterial wall). **(D)** Quantitative analysis of cell apoptosis in rats assessed by TUNEL staining. **(E)** The expression of apoptosis-related factors (cleaved Caspase-3, cleaved PARP1, Bax, and Bcl-2) as determined by Western blot analysis and quantified by the Image J software. *p < 0.05. The measurement data were expressed as mean ± standard derivation. Comparisons among multiple groups were analyzed by one-way ANOVA followed by Tukey’s *post hoc* test, and the cell experiment was conducted three times independently. The sham group: *n* = 12, the IA + sh-NC group: *n* = 12, and the IA + sh-MIAT group: *n* = 6.

Intracranial aneurysm model was successfully induced on SD rats, which were then intraperitoneally injected with viruses packaged with sh-MIAT (IA + sh-MIAT group) or sh-NC (IA + sh-NC group), with the sham group (without injection of viruses) as control. Four weeks after induction, RT-qPCR (Figure 5A) manifested that MIAT expression was increased obviously in rats after IA modeling (*p* < 0.05), and did not differ significantly in IA rats treated with sh-MIAT versus the sham-operated rats (*p* > 0.05). In IA rats, MIAT expression was decreased by sh-MIAT (*p* < 0.05). The sections of the anterior cerebral artery (ACA)/olfactory artery (OA) branch were obtained. HE staining revealed that IA rats showed thinner aneurysm wall than the sham-operated rats, which was recovered after treatment with sh-MIAT. In addition, endothelial cells were continuously aligned at the expected position of the artery, with no gap among these cells in sham-operated rats. In contrast, in IA rats, a small number of endothelial cells were lost occasionally, resulting in the disconnection of endothelial cells and thereby forming a gap (Figures 5B,C).

Simultaneously, TUNEL staining (Figure 5C) exhibited that apoptosis rate in rats after IA induction was elevated, which was attenuated by sh-MIAT. Meanwhile, the augmented cleaved Caspase-3, cleaved PARP1, and Bax expression but reduced Bcl-2 expression in rats after IA modeling determined by Western blot analysis also confirmed the presence of elevated cell apoptosis of rats after IA induction, which was reversed by silencing MIAT (Figure 5E). Collectively, MIAT silencing reduced the apoptosis rate of endothelial cells, which was conducive for maintenance of the continuity of endothelial cells and exerted certain preventive effects on the pathogenesis of IA in rats.

### Silencing of ENC1 Inhibited Endothelial Cell Apoptosis and Progression of IA *in vivo*

**FIGURE 6:**
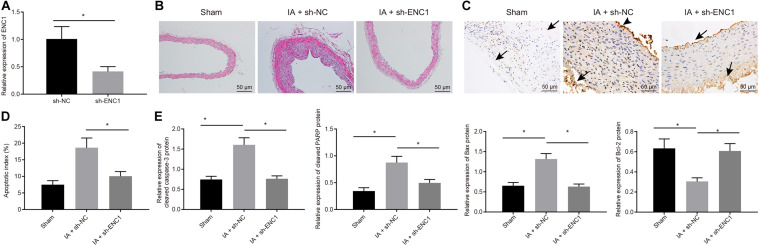
Silencing ENC1 reduces endothelial cell apoptosis *in vivo* to protect rats from IA. Rats were induced with IA model and treated with sh-ENC1 or sh-NC, with the sham-operated rats as control. **(A)** The expression of ENC1 in rats determined by RT-qPCR. **(B)** HE staining (×200) for the ACA/OA branch sections of rats. **(C)** The representative images of TUNEL staining (×200) for apoptosis of rats (arrows indicate the arterial wall). **(D)** Quantitative analysis of cell apoptosis in rats evaluated by TUNEL staining. **(E)** The expression of apoptosis-related factors (cleaved Caspase-3, cleaved PARP1, Bax, and Bcl-2) as determined by Western blot analysis and quantified by Image J software. *p < 0.05. The measurement data were expressed as mean ± standard derivation. Comparisons between two groups were analyzed by unpaired *t* test, and comparisons among multiple groups were analyzed by one-way ANOVA and followed by Tukey’s *post hoc* test. The cell experiment was conducted three times independently. The sham group: *n* = 12, the IA + sh-NC group: *n* = 12, and the IA + sh-ENC1 group: *n* = 6.

To further explore the role of ENC1 *in vivo*, IA rat models were induced, and then intraperitoneally injected with viruses packaged with sh-ENC1 (IA + sh-ENC1 group) or sh-NC (IA + sh-NC group), with sham group (without injection of viruses) as control. Decline of ENC1 expression in IA rats was caused by sh-ENC1 treatment, as measured by RT-qPCR (Figure 6A). Then HE staining revealed that, compared to the sham-operated rats, the blood vessels in IA rats treated with sh-NC were strikingly deformed, whilst in contrast to IA rats treated with sh-NC, the vascular lesions in IA rats treated with sh-ENC1 were improved (Figure 6B). TUNEL depicted that the TUNEL-positive rate in rats was enhanced after IA modeling, which was counteracted by sh-ENC1treatment (Figures 6C,D). Consistent results were revealed by western blot analysis (Figure 6E). The upregulated Cleaved caspase-3, cleaved PARP1, and Bax but downregulated Bcl-2 was observed in rats after IA modeling, which was abrogated by silencing ENC1 (*p* < 0.05). The aforementioned findings demonstrated that in the rat model of IA, ENC1 down-regulation could inhibit IA occurrence.

## Discussion

Intracranial aneurysm is an arterial anomaly affecting approximately 2% of the population worldwide and the rupture of IA consequences in high mortality. Recent studies have identified the oncogenic role of MIAT in several types of cancers such as papillary thyroid cancer and non-small cell lung cancer (Lin et al., 2019; Liu et al., 2019). However, the function of MIAT in IA remains elusive. Therefore, the current study intended to investigate the molecular mechanism of MIAT in IA, and our study demonstrated that MIAT knockdown inhibited the expression of ENC1 via MYC, thereby inhibiting the apoptosis of vascular endothelial cells *in vitro*, and ultimately repressing IA formation *in vivo*.

In the current study, a high expression of MIAT was validated in the blood sample of IA patients, especially in the ruptured IA samples. In consistency with our finding, it has been previously reported that MIAT was highly expressed in osteosarcoma tissues and silencing MIAT exercised an inhibitory effect on the progression of osteosarcoma (Jin et al., 2019a). To explore the function of overexpression of MIAT in IA, vascular endothelial cells were infected with oe-MIAT, which resulted in increased expression of cleaved Caspase 3, cleaved PARP1, and Bax along with decreased expression of Bcl-2. Moreover, Caspase-3, cleaved PARP1 (a substrate of caspase-3) and Bax (a Bcl-2 family protein) have been confirmed as vital indicators of apoptosis (Bressenot et al., 2009; Buschhaus et al., 2017; Ling et al., 2017; Reyna et al., 2017). In addition, MIAT silencing could evidently reduce cell apoptosis, which was conducive to maintaining the continuity of endothelial cells and ultimately had a preventive effect on the occurrence of IA in this study. Correspondingly, silencing MIAT could extensively inhibit the apoptosis of vascular endothelial cells in diabetic rats (Huo et al., 2019). Hence, the aforementioned findings supported the preventive property of MIAT knockdown in the pathogenesis of IA.

The subsequent finding in the current study was that MIAT increased the expression of ENC1 by promoting the ability of MYC binding to the promoter region of ENC1. Myelocytomatosis oncogene is a TF that plays multiple functions in several biological processes like proliferation and differentiation and is associated with various cancers such as lymphomas and breast cancer (Kleo et al., 2019; Lourenco et al., 2019). Similarly, another study documented that the transcriptional activity of MYC can be maintained by lncRNA MYMLR (Kajino et al., 2019). Interestingly, it has been documented that FOXO1 can regulate MYC to influence the angiogenesis during diabetic wound healing (Huang et al., 2019). However, in our study, we predicted that MIAT orchestrated MYC through the LncMAP database, which was validated by dual luciferase reporter assay, RIP assay, and RNA pull down experiment. These findings indicate that there are numerous molecular pathways in IA, and more mechanisms need further experimental exploration. This study further established that ENC1 was also highly expressed in IA patients, and ENC1 knockdown inverted the apoptosis induced by MIAT, consequently lowering the risk of IA occurrence. Ectodermal-neural cortex 1 is also overexpressed in adrenocortical adenomas (Durand et al., 2011). Besides, ENC1 knockdown can fundamentally result in inhibition of proliferation, migration, and invasion in ovarian cells (Fan et al., 2019), which was in compliance with our findings denoting the inhibitory property of ENC1 silencing in IA.

## Conclusion

**FIGURE 7:**
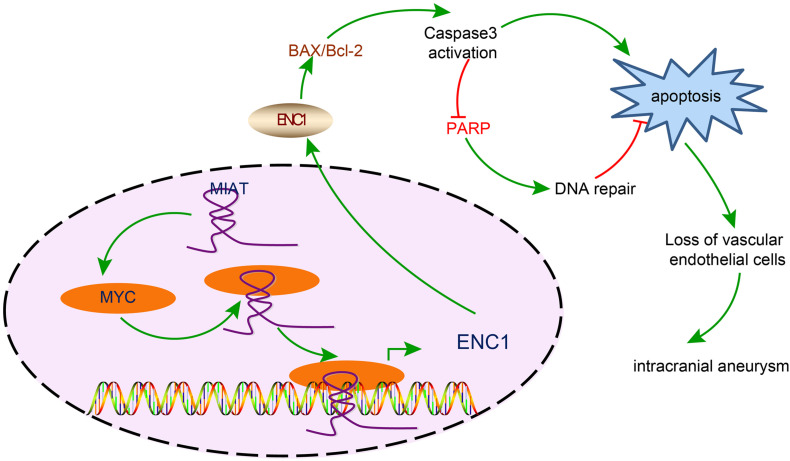
The mechanism diagram illustrating the effects of the MIAT/MYC/ENC1 axis on endothelial cell apoptosis in IA. MIAT enhanced the expression of ENC1 through MYC, thereby promoting vascular endothelial cell apoptosis and further inducing the pathogenesis of IA.

In summary, this study provided evidence demonstrating that the silencing of MIAT downregulated ENC1 by suppressing the transcription activity of MYC, thus reducing the apoptosis of vascular endothelial cells, refining endothelial cell continuity and lowering the incidence of IA (Figure 7). Based on the evidence from this study, therapeutic strategies should essentially be directed toward the down-regulation of MIAT, which may be a potentially viable target for the treatment of IA in the future.

## Data Availability Statement

The original contributions presented in the study are included in the article/Supplementary Material, further inquiries can be directed to the corresponding author/s.

## Ethics Statement

The studies involving human participants were reviewed and approved by the Ethics Committee of the First Hospital of China Medical University. The patients/participants provided their written informed consent to participate in this study. The animal study was reviewed and approved by the Ethics Committee of the First Hospital of China Medical University.

## Author Contributions

HZ and JT wrote the manuscript. XL and HZ performed the research. XL and JL analyzed the data. JL and JT contributed new reagents/analytical tools. All authors designed the research, contributed to the revision of the manuscript, and approved the final version.

## Conflict of Interest

The authors declare that the research was conducted in the absence of any commercial or financial relationships that could be construed as a potential conflict of interest.

## Footnotes

1 https://www.ncbi.nlm.nih.gov/geo/

2 http://www.bioconductor.org/packages/release/bioc/html/limma.html

3 http://lncatlas.crg.eu/

4 http://bio-bigdata.hrbmu.edu.cn/LncMAP/

5 https://string-db.org

6 https://cytoscape.org
