# Supplementary material for: Long Non-coding RNA MIAT Knockdown Prevents the Formation of Intracranial Aneurysm by Downregulating ENC1 via MYC
Source: Front Physiol. 2021 Jan 21;11:572605. doi: 10.3389/fphys.2020.572605 (PMC7860976; doi:10.3389/fphys.2020.572605)
Supplement: Supplementary file 1 [file Table_1.DOCX]

Supplementary Table 1

| GSE75436 AND lncMAP | ENC1 |
| --- | --- |
| lncMAP | TRIP6、YWHAG、GDA、HTR4、CASP6、PARP12、NCS1、STX1A、PPP3CA、KCNAB2、SLC26A8、BDNF、PI4KA、STEAP3、DNM1、CAMK2A、NRGN、PDP1、PNMAL2、SLC47A2、CDKN2D、GLS、TEAD2、BRSK1、NAPB、SGTB、PPIC、SHISA7、MICAL1、SLC27A2、PTER、BCL11A、USP43、REM1、PRRT2、SH2D5、FBXL2、SCN5A、ANXA5、DRG2 |
| GSE75436 | HPSE2、SLMAP、CDH3、NPY5R、RYR3、CTD-2165H16.3、ABRA、PTN、AF070581、SDS、LINC00643、ADRA2C、BLNK、GFRA3、VAMP8、CXCR4、SALL1、CRTAM、ADAM12、CXCL14、CD163、SULT1E1、DPT、LDB3、CHRDL1、SORCS1、KRT14、PPP1R14A、SUSD5、HMOX1、HOXD8、SYNDIG1、WISP2、ZDHHC11、GPIHBP1、FBXO16、KCNT2、MFAP4、C1QC、CECR1、RASSF3、CILP、GZMH、C15orf48、TYROBP、GPR65、NLGN4X、LGR6、TMEM158、SFRP5、EBI3、TNC、NETO2、XK、DSP、ITLN1、PDE2A、BAI3、CCL18、FPR3、SYNPO2、TSPAN12、PRAM1、SLC12A8、SHISA6、FAP、ALOX5、NR2F1、RNASE6、TUSC5、LEP、CCR1、MPZ、LHFPL2、SDSL、GPR83、PLEK2、MYOM1、CD33、IRX5、LINC00950、NAPSB、IGSF10、GPR20、MYO3A、ENTPD3、C5AR1、IFI6、ADAM33、KIAA1644、BVES-AS1、BTC、FAM46B、ACP5、HIGD1B、LGMN、CNN1、SLCO2B1、PLCB4、LINC00460、SIGLEC9、LILRB1、TLR1、ZIC2、ALOX5AP、PLN、POSTN、MEOX1、INA、MOV10L1、SYNGR3、ADAMDEC1、SLC16A6、RFPL1S、TRDN、SCG2、ARHGEF26-AS1、CARNS1、MPP7、FAM150B、TREM2、MS4A4A、CTD-3064M3.3、ACKR1、RERG、DAB1、CCL19、UTS2、ANXA13、RASL12、GALNT6、COL28A1、PPFIA2、CPXM1、FFAR4、PPAPDC1A、CHST9、NEFH、LAIR1、EVI2A、PRIMA1、PLA2G2A、CNTN4、ROR2、CHI3L2、ZNF385B、MAOA、IFNK、APOE、RBM24、VAT1L、TNNT3、CTB-12O2.1、SDK1、SDC1、PRDM16、CYS1、PMP2、SEMA3E、FNDC1、ERP27、BCHE、TSPAN8、CKB、LOC101928076、INSC、LOC101929122、KLRG2、TNFRSF11B、C3AR1、CADM2、CEMIP、ZBED9、NME8、C12orf75、GPR34、LPAR5、SASH3、PPP1R9A、FAXC、FBXL22、HOXD-AS2、BCAM、EMX2、APOD、LGI1、EN1、ANGPTL7、CRYBB1、NCKAP1L、RGS4、SLC13A4、C16orf89、AVPR1A、WNT11、KLRC3、FYB、NRG3、F10、MYOC、RNASE2、TMEM52B、TMEM35、ADORA3、MRGPRF、LY86、IGSF11、APOBEC2、TNFRSF21、ACTC1、DLX5、LINC01094、OXTR、ITIH2、CORIN、SCN4A、CDH2、NEXN、TPO、LINC01116、LINC00889、FLNC、PMAIP1、MYOT、CADM3-AS1、ARL4C、PCDH8、RP11-320H14.1、MAL、NTF3、LOC100506119、CXCL1、PC、PDZRN4、FCGBP、FRK、PLA2G7、KRT18、RYR2、LL22NC03-N14H11.1、MT1M、TNNC1、C1QB、OSR2、PKIB、COLEC11、ALX3、HOXD4、SIGLEC10、FCGR1B、SOX2-OT、VMO1、CXCL8、KCNA5、KIAA1755、P2RX1、DCSTAMP、CLDN7、ADIRF、GZMK、LRRC25、SELE、HK3、CRMP1、VSTM2A、GPR84、RSPO2、EMR1、MLIP、ADORA2B、MS4A6A、LOC101929133、DLGAP5、IP6K3、AP000525.9、OTOGL、C4orf48、PAIP2B、SYBU、SMTN、FLJ41170、BTK、CD180、CA3、COL4A6、AQPEP、SCN7A、PGM5-AS1、NRIP2、LOC101929335、TMEM163、TFEC、MS4A7、CXCL10、CCL21、CAPN6、SCN3A、ITIH1、PPL、RRAD、KCNJ6、VIT、CCL26、SERPINE1、LMO3、VCAN、KLHL6、EYA1、ADAP2、SLC46A2、AOC3、FABP3、RORB、SLC35F1、GPR158、PACSIN3、MAGEL2、WFDC21P、CSRP1、CTSB、OR51E2、TNF、FGFBP2、CHL1、APBA2、RGS6、TTLL7、FGF13、P2RX5、COBL、SLA、PLEKHG3、CPVL、CAV3、MEST、IL32、LINC00607、COL5A2、CD300LF、LMOD1、SFTA1P、METTL7B、SLC6A12、KIRREL3、DES、LYZ、ASPA、RARRES1、HOTS、HLA-DMB、CLEC5A、CSF3、AK5、LAPTM5、FABP4、CSRP2、UNC13C、KCNE2、C5orf46、SOSTDC1、CAB39L、SBSPON、HRC、LOC101929759、ENPP6、PCP4、GPC3、GRP、CXCL6、KYNU、MPEG1、CD8A、ART3、CCR5、CCL20、TNMD、SLAMF8、SPP1、GLI1、IL17B、FCGR1A、STAC、PPP1R1A、FZD3、WNT5A、MYH11、CDH19、ALDH1B1、PGM5、C1QTNF7、EYA4、KIF20A、OLR1、NOV、EMX2OS、PCK1、GPM6B、KMO、CIDEA、SH3BGR、IL21R、HAS2、SOX11、IL1B、ARSE、ACTA1、MARCO、IL18、MBNL1-AS1、IGSF6、IGFBP2、CRLF1、SLCO5A1、SIGLEC1、ATP8A2、CCL4、MSR1、PPP1R14C、COL10A1、FCER1G、LAMC3、NPY1R、WNT4、SOST、TAC1、CYP4F12、GKAP1、SFRP4、SHOX2、ITGA7、VSIG4、RP3-525N10.2、AGTR1、MCAM、ANO3、ITIH3、HAPLN2、SEL1L2、PLAC8、FREM1、C1QA、HAMP、KCNAB1、MELK、PLVAP、DPY19L2、COL11A1、DOCK3、LOC100507403、FOXF2、LINC00702、LINC00982、CASQ2、GAP43、CKMT2、PYGM、NPNT、C10orf82、TIMP4、ITGB2、C2orf88、PRUNE2、CD72、PLP1、PGAM2、CTHRC1、TLR2、SIGLEC7、WFDC1、LOC100505774、HN1、APOL1、PTCHD1、BCAT1、LOC101929340、KBTBD12、SLC7A11、FHL5、FOXD3-AS1、LRRTM1、RELN、ZIC3、KANK4、XIRP2、RBPMS2、GREM1、LRRC10B、RP11-375I20.6、XKR4、IGFBPL1、NRXN1、NTS、C3orf70、RBP1、CXCL16、RXFP1、MIAT、EGFEM1P、MIR143HG、IGFL2、SULT1B1、HAVCR2、MIA、TMEM56、CCDC178、PCSK2、GAD1、ZG16B、MYLPF、BC015159、SCG5、NUF2、CTNNA3、CD86、SLITRK6、SCGB3A2、C2orf40、CP、OCA2、FREM2、SERPIND1、AMIGO2、DAPL1、ANKRD34C、KCNJ8、IBSP、AMPD3、LOC100507461、CA12、CFD、APOC1、EPHX2、RERGL、FLVCR2、LPHN3、CYP4B1、REEP1、UBE2T、MYOCD、DUXAP10、MS4A14、LOC102723918、SLC16A10、BCL2A1、ANGPTL1、KLHL32、LOC100507165、FHOD3、SRL、PTCH2、LINC00632、SLC22A3、ALPK2、GAPT、ASB2、LINC00310、SPOCD1、LAMP5、CCRL2、FPR1、PLIN1、CD14、LINC00844、ADIPOQ |
